# Ultrasound-guided thrombin injection for the treatment of an iatrogenic hepatic artery pseudoaneurysm: a case report

**DOI:** 10.1186/1752-1947-5-518

**Published:** 2011-10-21

**Authors:** Hiroyuki Tokue, Yoshito Takeuchi, Ketaro Sofue, Yasuaki Arai, Yoshito Tsushima

**Affiliations:** 1 Department of Diagnostic and Interventional Radiology, Gunma University Hospital, Maebashi, Gunma, Japan; 2 Division of Diagnostic Radiology, National Cancer Center Hospital, Tokyo, Japan

## Abstract

**Introduction:**

Percutaneous transhepatic portal embolization is often performed to expand the indications for hepatic resection. Various etiologies of hepatic artery pseudoaneurysm have been reported, but regardless of the etiology, hepatic artery pseudoaneurysm is usually managed with an endovascular approach or open surgery, depending on the location and clinical symptomatology. However, it is difficult to manage hepatic artery pseudoaneurysm after percutaneous transhepatic portal embolization, since embolization of the hepatic artery may cause hepatic infarction

**Case presentation:**

A 58-year-old Japanese man with hilar bile duct cancer underwent percutaneous transhepatic portal embolization to expand the indication for hepatic resection. Two days after percutaneous transhepatic portal embolization, our patient suddenly complained of abdominal pain. Contrast-enhanced computed tomography confirmed a pseudoaneurysm arising from a segmental branch of his right hepatic artery. Since embolization of the hepatic arterial branches may cause hepatic infarction, ultrasound-guided thrombin injection therapy was successfully performed for the pseudoaneurysm.

**Conclusion:**

We performed a thrombin injection instead of arterial embolization to avoid hepatic infarction. The rationale of this choice may be insufficient. However, ultrasound-guided percutaneous thrombin injection therapy may be considered as an alternative to percutaneous transarterial embolization or surgical intervention for an iatrogenic hepatic artery pseudoaneurysm.

## Introduction

Percutaneous transhepatic portal embolization (PTPE) is often performed to expand the indications for hepatic resection. Various etiologies of hepatic artery pseudoaneurysm (HAP) have been reported, but regardless of the etiology, HAP is usually managed with an endovascular approach or open surgery, depending on the location and clinical symptomatology. However, it is difficult to manage HAP after PTPE, since embolization of the hepatic artery may cause hepatic infarction. We herein describe a case of PTPE complicated by a HAP, in which the HAP was successfully managed with an ultrasound (US)-guided thrombin injection technique.

## Case presentation

A 58-year-old Japanese man with hilar bile ductal carcinoma underwent preoperative PTPE to expand the indication for right hepatic resection. We punctured the anterior branch of his right portal vein with a 21-gauge needle under US-guidance. A 5-Fr sheath was advanced into the portal branch and a 5-Fr balloon catheter was inserted into the anterior and posterior branches of his right portal vein. After inflating the balloon, absolute alcohol was injected. A portography confirmed the complete occlusion of these portal branches. Finally, two 5 mm × 5 cm 0.035-inch coils were deployed to perform tract embolization after PTPE. During these procedures our patient was asymptomatic.

**Figure 1:**
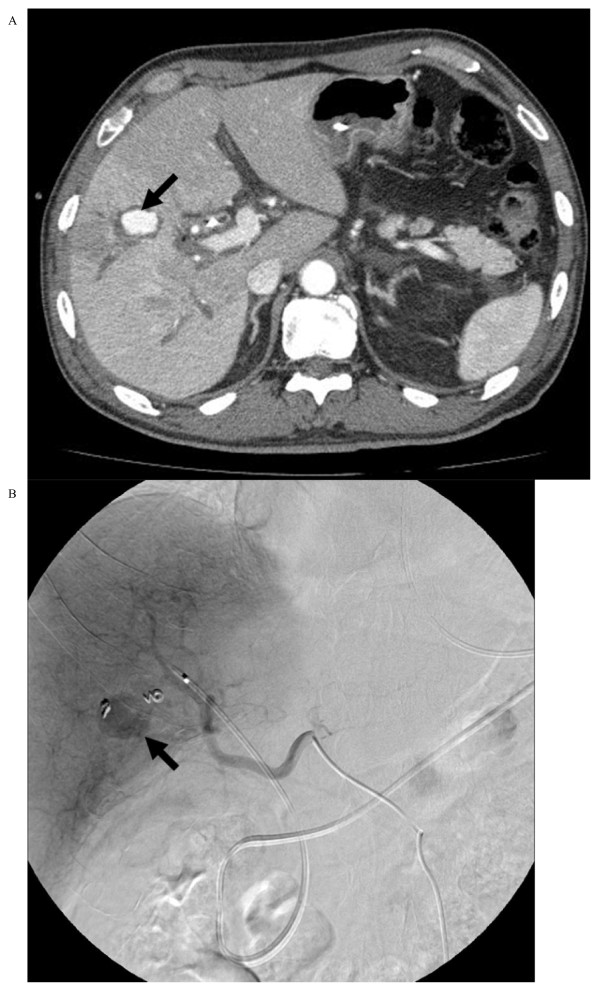
**Pseudoaneurysm (arrow) arising from the right hepatic artery branch**. **(a) **Contrast enhanced CT of the upper abdomen. **(b) **DSA of the proper hepatic artery.

**Figure 2:**
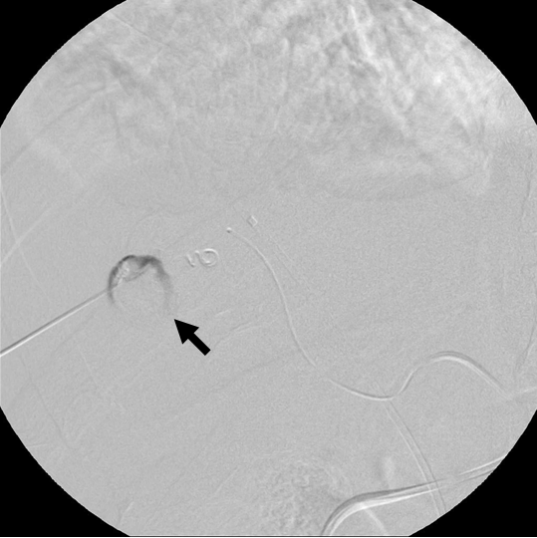
**US-guided thrombin injection therapy for an iatrogenic hepatic artery pseudoaneurysm with 21G needle (arrow)**.

**Figure 3:**
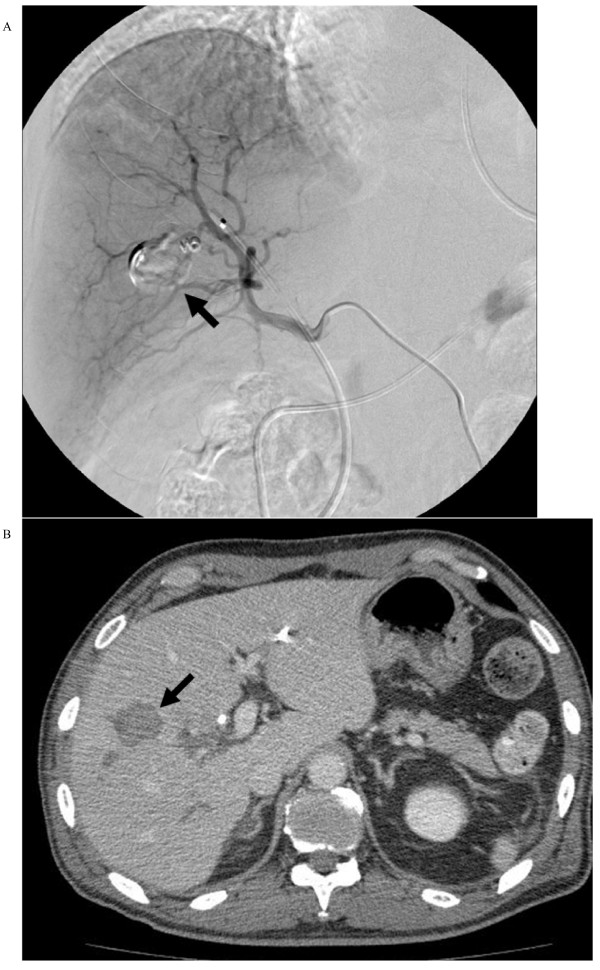
**Confirmation of the total occlusion of the pseudoaneurysm on (a) DSA and (b) follow-up CT**.

Two days later, our patient suddenly complained of an acute abdominal pain, but his vital signs remained stable. A contrast-enhanced computed tomography (CT) confirmed the presence of a pseudoaneurysm arising from a segmental branch of his right hepatic artery: the pseudoaneurysm measured 20 × 15 mm in size with a narrow neck surrounded by hematoma (Figure 1a). Percutaneous transarterial embolization (TAE) of the pseudoaneurysm was considered to be inappropriate, since TAE may cause hepatic infarction because of an already occluded portal vein. Under US and digital subtraction angiography (DSA) guidance (Figure 1b), the pseudoaneurysm was punctured with a 21-gauge needle and 1500 U of human-derived thrombin was injected into the pseudoaneurysm (Figure 2). Total occlusion of the pseudoaneurysm was confirmed by DSA and follow-up CT (Figure 3), and an occlusion of the segmental branch of his right hepatic artery was avoided. Our patient was followed-up for four weeks after the procedure using US, and there was no evidence of recurrent pseudoaneurysm or hepatic infarction. The left lobe of his liver became hypertrophic. He underwent a right hepatectomy 30 days after the procedure, and his postoperative course was uneventful.

## Discussion

Post-traumatic HAP is uncommon, and accounts for approximately 1% of hepatic trauma cases [1,2]. Other causes include chronic pancreatitis, orthotopic liver transplantation, arteriosclerosis, cystic medial necrosis, polyarteritis nodosa, necrotizing vasculitis, acute pancreatitis and hepatocellular carcinoma [2]. Most HAPs occur extrahepatically, predominantly in the right hepatic artery [2]. Intrahepatic HAPs account for only about 20% of all HAPs and are often a complication of percutaneous procedures such as transhepatic cholangiography, transhepatic catheter placement or liver biopsy [3]. The incidence of intrahepatic HAP occurring after trauma is relatively uncommon.

There is only one report of PTPE complicated by HAP, and it occurred in one of 47 procedures (2.1%) [4]. However, to the best of our knowledge, there have been no reports in the English literature describing treatment of HAP complicated by PTPE. In the present case, we suspected that the HAP may have been caused by unexpected damage of the hepatic arterial branch when we accessed his right portal vein. Rupture of a HAP is associated with a high mortality rate, thus it mandates an early detection and prompt intervention [1,2]. Although clinical diagnosis can be made by noninvasive methods such as CT and Doppler US, selective catheter arteriography remains the most sensitive modality for detecting a HAP. In a study by Tobben *et al*. [5], catheter arteriography detected all HAPs in ten patients, compared with only 67% by CT and 33% by Doppler US. Selective arteriography may also show active bleeding and anatomic variations such as an anomalous or replaced hepatic artery [6], and can be used in simultaneous diagnosis and treatment. The recent extended utilization of high-resolution vascular imaging modalities may have a greater contribution.

Selective arterial embolization is currently considered to be the most appropriate technique in the treatment of visceral pseudoaneurysms, with a success rate of more than 80% and a low complication rate [7]. Various agents for embolization have been used successfully, such as ethanol, gel foam particles, microcoils, n-butyl-2-cyanoacrylate glue, polyvinyl alcohol particles and thrombin [8,9] as well as metallic stents and detachable silicone balloons [10].

Percutaneous thrombin injections for the treatment of visceral [11], renal [12] and extremity pseudoaneurysms have been employed since 1986 and were first described by Cope and Zeit [13], and can be performed under Doppler US-guidance. This method has yielded excellent results for femoral pseudoaneurysms, and can be carried out without the need of anesthesia equipment or an operating theater. We selected the percutaneous thrombin injection technique under US and DSA-guidance to avoid hepatic artery occlusion which may result in hepatic infarction.

As well as the possibility of a recurrent pseudoaneurysm after a percutaneous thrombin injection, complications such as thromboembolism and allergic reactions have limited its use [13]. The use of bovine-derived thrombin may pose a potential risk of an allergic response and hemorrhage in patients with a known allergy to bovine-derived products or previous exposure to topical thrombin [13]. Another consequence of bovine thrombin exposure is the potential development of antibodies to human clotting proteins and thrombin, in particular factor V, resulting in coagulopathy and excessive bleeding [14]. Such complications are not seen with newer human-derived thrombin.

## Conclusion

A HAP is one of the possible complications following PTPE. Generally, such a complication will be managed by an endovascular approach. Although minor hepatic infarction can occur after hepatic arterial embolization, liver damage induced by hepatic arterial embolization in such cases may usually be within an acceptable range. We performed thrombin injection instead of arterial embolization to avoid hepatic infarction. The rationale for this choice may be insufficient. However, US-guided percutaneous thrombin injection therapy may be considered as an alternative to TAE or surgical intervention for an iatrogenic HAP.

## Consent

Written informed consent was obtained from the patient for publication of this case report and any accompanying images. A copy of the written consent is available for review by the Editor-in-Chief of this journal.

## Competing interests

The authors declare that they have no competing interests.

## Authors' contributions

HT reviewed relevant literature and drafted the manuscript. All authors provided clinical expertise and participated in drafting the manuscript. And all authors read and approved the final manuscript.
